# DFT Meets Wave-Function Composite Methods for Characterizing Cytosine Tautomers in the Gas Phase

**DOI:** 10.1021/acs.jctc.3c00465

**Published:** 2023-07-21

**Authors:** Vincenzo Barone

**Affiliations:** Scuola Normale Superiore di Pisa, Piazza dei Cavalieri 7, 56126 Pisa, Italy

## Abstract

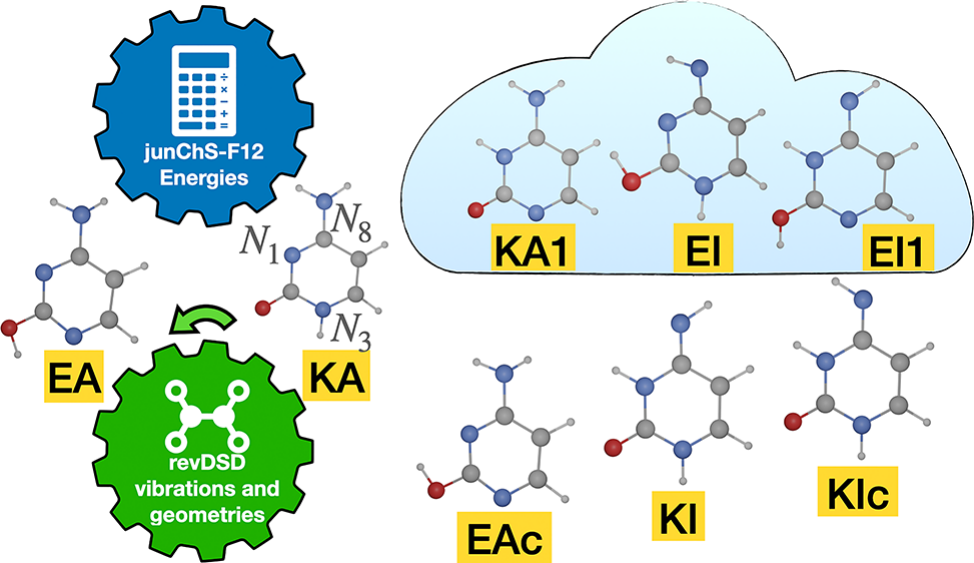

A general strategy for the accurate computation of structural and spectroscopic properties of biomolecule building blocks in the gas phase has been further improved and validated with a special reference to tautomeric equilibria. The main improvements concern the use of the cc-pVTZ-F12 basis set in both DFT and CCSD(T)-F12 computations, the inclusion of core-valence correlation in geometry optimizations by double hybrid functionals, and the use of the cc-pVQZ-F12 basis set for complete basis set extrapolation at the MP2-F12 level. The resulting model chemistry is applied to the challenging problem of cytosine tautomers in the gas phase. The results are in remarkable agreement with experiment concerning both rotational and vibrational spectroscopic parameters and permit their unbiased interpretation in terms of structural and thermochemical features. Together with the intrinsic interest of the studied molecule, the accuracy of the results obtained at reasonable cost without any empirical parameter suggests that the proposed composite method can be profitably employed for accurate investigations of other molecular bricks of life.

## 1 Introduction

Understanding the structure of the DNA and RNA nucleobases is fundamental to modeling and predicting their behavior in larger molecules, such as nucleosides, nucleotides, and DNA itself. Several tautomeric forms are, in principle, possible for each nucleobase, and it was recognized very early that tautomerism can lead to mutations. The number of possible tautomers depends on the number of labile protons and the number of conjugated tautomeric sites. Furthermore, the relative stability of individual tautomers is strongly tuned by various internal and external factors,^1,2^ with the keto- and amino-forms predominating under physiological conditions. As a consequence, these species are considered as “major” or “canonical” tautomers, whereas the enol- and imino-forms are considered as “rare” or “minor” tautomers. Systematic investigations have revealed that the canonical forms of uracil,^3−5^ thymine,^6^ and adenine^7^ prevail in the gas phase, in solution, and in the solid state. However, the presence of some tautomers with comparable relative stability, but strongly different dipole moments, leads to dramatical changes of the relative populations for different tautomers of cytosine and guanine when going from gas phase (or inert matrices) to aqueous solution (or solid-state). Therefore, the disentanglement of the role played by different contributions in tuning the tautomeric equilibrium of cytosine and guanine requires a preliminary analysis of their behavior in the gas phase or in inert matrices.^8^

The tautomeric equilibrium of guanine in the gas phase has been recently investigated by microwave spectroscopy^9^ and by state-of-the-art quantum chemical (QC) methods.^10^ The latter study confirms the strong sensitivity of the results to the level of computation whenever the energy difference between some tautomers amounts to a few hundreds of cm^–1^ (i.e., 4–5 kJ/mol). Since state-of-the-art computations have not yet been performed for the tautomeric equilibrium of cytosine, which is even more complex than guanine, this system was studied by a computational strategy, which has been recently devised for the accurate computation of structures and spectroscopic parameters for flexible molecules containing few dozen atoms belonging to the first three rows of the periodic table.^11−14^

From an experimental point of view, different spectroscopic studies detected two or three cytosine tautomers.^15−22^ Matrix isolation (argon and nitrogen matrices at 15 K) infrared (IR)^15^ and molecular beam microwave (MW)^16^ spectroscopy suggested the presence of a mixture of the keto-amino tautomer and the “rare” enol-amino and keto-imino tautomers, while through a resonance-enhanced multiphoton ionization (REMPI)^17^ investigation, it was established that both keto-amino and enol-amino tautomers coexist in the gas phase. The rotational constants of three cytosine tautomers identified by MW spectroscopy^16^ have been correlated with the keto-amino, keto-imino, and enol-amino tautomers. Cis and trans rotamers of the enol-amino tautomer were identified in helium nanodroplets at 0.37 K together with the keto-amino form.^18^ In experiments in an argon matrix, photoisomerization processes induced by narrowband tunable near-IR^20^ and ultraviolet (UV) (λ = 311 nm)^21^ light were interpreted in terms of the coexistence of the same three tautomers detected in ref (16). Finally, Alonso et al.,^22^ using laser-ablation molecular-beam Fourier-transform microwave (LA-MB-FT-MW) spectroscopy, identified five species of gaseous cytosine, namely the keto-amino form together with two rotamers each for the enol-amino and keto-imino forms.

From a theoretical point of view, most QC calculations predicted that there are small energy differences between the lowest energy tautomers of cytosine with the quantitative values being extremely sensitive to the theory level.^23−29^ However, even the most refined computations performed till now^28^ did not take into account complete basis set (CBS) extrapolation or core valence (CV) correlation. Furthermore, the limited accuracy of the geometries employed for single-point energy evaluations does not allow a fully unbiased comparison with the rotational constants issued from experiments. Finally, zero point energies (ZPEs) and thermal contributions (TCs) to free energies have been computed within the rigid-rotor harmonic-oscillator (RRHO) model with low-level QC methods.

As already mentioned, an integrated computational approach has been recently developed, which combines different QC methods for an effective exploration of the potential energy surfaces (PES) of biomolecule building blocks and the successive refinement of the most significant stationary points.^11,12,30^ In this framework, once a suitable panel of low-energy minima has been defined, accurate structures are obtained by refining the optimized geometries by a linear regression approach (LRA),^31,32^ and accurate relative energies are computed by composite wave-function methods.^33−38^ Finally, ZPEs and TCs contributions to thermodynamic functions and spectroscopic parameters of the energy minima with non-negligible populations under the experimental conditions of interest are computed at the DFT level.^39^

This procedure has been validated for several amino acids containing polar or aromatic side chains.^13,40−42^ However, the presence of topological changes (i.e., different bonding patterns) in the different tautomers might require QC methods even more accurate than those validated for the analysis of conformer stabilities. Here this problem is tackled for the particularly challenging case of cytosine in the gas phase, with special reference to the MW and IR spectra of the different tautomers. As already mentioned, previous studies of this molecule employed QC methods of limited accuracy and payed marginal attention to the geometrical parameters, with these limitations allowing at most a posteriori interpretations in terms of the agreement between experimental and computed spectroscopic parameters for a predefined number of different species. The present approach allows, instead, an unbiased comparison with spectroscopic results thanks to the accuracy of the computational results.^14,34,43^

## 2 Computational Details

On the basis of previous experience, the first characterization of PESs has been performed at the B3LYP/6-31+G* level,^44,45^ also including Grimme’s D3BJ dispersion corrections.^46^ In the following, this combination of functional and basis set, which is used also for the computation of anharmonic contributions, will be referred to simply as B3. Next, the geometries and harmonic force fields of the most stable species have been refined at the revDSD-PBEP86-D3BJ/jun-cc-pvTZ level^47−50^ (hereafter rDSD/j3). Several studies have shown that neglect of diffuse *f* functions on non-hydrogen atoms (which leads from the jul-cc-pVTZ to the jun-cc-pvTZ basis set) reduces the computational cost without any significant accuracy loss for geometries,^32^ dipole moments,^51^ and spectroscopic parameters.^39,52^

The typical error of rDSD/j3 bond lengths and valence angles (0.3%)^32^ is sufficient to obtain accurate relative electronic energies and vibrational spectra of different tautomers, but the situation is different for the leading terms of MW spectra, namely rotational constants of the vibrational ground-state (*B*_*i*_^0^, where *i* refers to the inertial axes *a*, *b*, *c*). These parameters include vibrational corrections (Δ*B*_*i*_^vib^) in addition to equilibrium rotational constants (*B*_*i*_^eq^).^53^ Since the experimental determination of vibrational corrections is practically impossible except for very small molecules, their computation by QC methods (leading to the so-called semi-experimental (SE) equilibrium rotational constants *B*_*i*_^SE^) has emerged as a very accurate alternative.^53,54^ Actually, affordable levels of theory (B3 in the present context) can be employed to evaluate the Δ*B*_*i*_^vib^ contributions since their values are typically well below 1% of the corresponding *B*_*i*_^eq^ rotational constants.^55^ For small molecules, isotopic substitutions produce a sufficient number of SE equilibrium rotational constants to allow the determination of very accurate SE equilibrium geometries by non-linear least-square fittings.^56^ This approach cannot be followed for larger molecules due to the lack of experimental data, but the quality of QC equilibrium geometries can be estimated from the comparison of the corresponding equilibrium rotational constants with their SE counterparts for the main isotopologue. In this framework, the average errors of state-of-the-art QC methods for the rotational constants of small semi-rigid molecules are of the order of 0.1%,^57,58^ whereas the average errors of the standard methods employed in the analysis of MW spectra of biomolecule building blocks are of the order of 1%. We have recently shown that the systematic nature of the errors permits to improve significantly the rDSD/j3 geometrical parameters and thus equilibrium rotational constants, by a LRA.^32,59,60^ Despite its remarkable success, the use of empirical parameters in the LRA is not fully satisfactory. As a consequence, in the present paper, the new Pisa composite scheme (PCS)^61^ will be employed, which takes into account CV correlation and does not require any empirical parameter.

Since cytosine contains three ^14^N quadrupolar nuclei (N1, N3, and N8), nuclear quadrupole coupling constants (χ_*ii*_, with *i* referring to the inertia axis *a*, *b,* or *c*) are important for accurate predictions of rotational spectra because they determine a splitting of the rotational transitions, which generates the so-called hyperfine structure.^57,62^ Furthermore, the components of dipole moments (μ_*i*_) determine the intensities of rotational transitions.^62,63^ Several studies have shown that dipole moments and quadrupole coupling constants can be computed with sufficient accuracy at the rDSD/j3 level.^13,51^ On the other hand, accurate electronic energies can be obtained by single-point energy evaluations using composite wave function methods as described in the next section.

The relative stability of different tautomers is determined by the corresponding relative enthalpy at 0K (Δ*H*_0_^°^) or free energy (Δ*G*°) at a temperature depending on the experimental conditions. The contribution of small amplitude vibrations to the differences between the thermodynamic functions of different tautomeric forms can be computed with sufficient accuracy by the harmonic oscillator model. While the amino forms of cytosine have a large amplitude vibration (governing the NH_2_ inversion), the corresponding harmonic frequency is relatively high (between 250 and 350 cm^–1^), so that the harmonic treatment provides sufficiently reliable results also for the contribution of this mode to thermodynamic functions. On the other hand, IR spectra have been computed without resorting to any scale factor in the framework of second-order vibrational perturbation theory (VPT2)^3,64−66^ and employing rDSD/j3 harmonic and B3 anharmonic contributions.^50,67,68^

All the conventional computations have been performed with the Gaussian package,^69^ with a sample input for performing PCS computations provided in the Supporting Information (SI). The Molpro^70^ software has been used, instead, for explicitly correlated computations employing the default options for auxiliary basis sets, complementary auxiliary basis sets (CABS), and geminal exponents.

## 3 Results and Discussion

As anticipated in the preceding section, the first point to be analyzed is the accuracy of the geometries delivered by different methods in order to obtain reliable rotational constants. In previous works, the rDSD functional in conjunction with the j3 basis set was employed with remarkable success. However, it is well known that converged results can be obtained by double-hybrid functionals only using (partially) augmented quadruple-zeta basis sets. Starting from this level, several test computations showed that *g* and diffuse *d,f* functions on non-hydrogen atoms together with diffuse and *f* functions on hydrogen atoms produce negligible changes in the geometrical parameters with a considerable saving of computer time. As a matter of fact, the cc-pVTZ-F12 basis set (hereafter 3F12), purposely developed for explicitly correlated computations,^71^ offers a nearly optimal cost/accuracy compromise also in conjunction with double-hybrid functionals. The resulting combination of functional and basis set (referred to in the following simply as rDSD) is taken here as the new standard for geometry optimizations. However, at these levels of accuracy, CV correlation cannot be neglected. Therefore, by adopting the same recipe employed in the ‘cheap’ family of composite methods,^5,30,34^ CV contributions are evaluated from the difference between all electron (*ae*) and frozen core (*fc*) MP2 computations performed with the cc-pCVTZ basis set^72^ (hereafter C3). The resulting PCS model^61^ leads to the following version of the so-called “gradient scheme” introduced by Gauss and co-workers^73,74^ for geometry optimizations by composite methods:
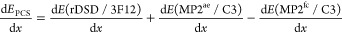
1

Since this approach would require considerable modifications of standard electronic structure codes, one can resort to the so-called “geometry scheme”^5^ in which the additivity approximation is directly applied to each geometrical parameter (r), i.e.,
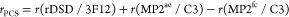
2

In this case, three different geometry optimizations are performed separately and then combined together. The close correspondence of geometrical parameters obtained by the two approaches has been proven several times.^5,75^ Since the size of the C3 basis set is comparable to that of its 3F12 counterpart, the computation of CV contributions can triple the computer time. However, several test computations showed that this increase is accompanied by a remarkable gain of accuracy. For purposes of illustration, Table 1 reports the rotational constants computed at different levels for uracil, which show unambiguously the remarkable accuracy of the PCS model. In structural terms, comparison with the SE geometrical parameters^76^ (see Table S1 of the SI) shows that the unsigned average error of bond lengths is reduced from 0.003 to 0.002 and 0.001 Å when going from rDSD/j3 to rDSD and PCS levels. At the same time, valence angles are always sufficiently accurate. As a matter of fact, the quality of the PCS results approaches that of state-of-the-art wave-function composite methods,^14,43^ but is reached with a huge reduction of computational cost, while still avoiding the use of empirical parameters. Noted is that, pending further tests, the rDSD/j3 model will be employed for the computation of harmonic frequencies and one-electron properties.

**Table 1:** Equilibrium Rotational Constants of Uracil and Amino Forms of Cytosine (KA, EA, and EAc) Obtained by Different Methods

	parameter	SE^a^	rDSD/j3	rDSD	PCS	B3LYP
uracil	B_*a*_^*eq*^	3913.3	3895.2	3901.8	3913.3	3875.9
	B_*b*_^*eq*^	2035.2	2022.2	2026.8	2033.3	2010.9
	B_*c*_^*eq*^	1338.9	1331.2	1333.9	1338.1	1324.0
	MUE		10.4	8.3	0.9	25.5
	MAX		18.1	11.5	1.9	37.4
	MUE%		0.63	0.36	0.05	1.09
	MAX%		0.56	0.41	0.09	1.19
cytosine (KA)	B_*a*_^*eq*^	3899.0	3884.8	3890.5	3904.3	3850.9
	B_*b*_^*eq*^	2034.4	2025.9	2029.7	2035.9	1992.1
	B_*c*_^*eq*^	1338.2	1331.9	1334.3	1338.5	1312.9
	MUE		9.7	5.7	2.4	29.9
	MAX		14.2	8.5	5.3	48.9
	MUE%		0.42	0.25	0.08	1.27
	MAX%		0.47	0.29	0.14	1.23
cytosine (EA)	B_*a*_^*eq*^	3979.5	3963.8	3970.4	3984.7	3935.4
	B_*b*_^*eq*^	2020.0	2011.3	2014.6	2020.9	1996.1
	B_*c*_^*eq*^	1341.1	1334.9	1337.1	1341.5	1324.9
	MUE		10.2	6.3	2.2	28.1
	MAX		15.7	9.5	5.2	44.1
	MUE%		0.43	0.27	0.07	1.17
	MAX%		0.46	0.30	0.13	1.21
cytosine (EAc)	B_*a*_^*eq*^	3920.1	3898.4	3904.1	3917.3	3870.1
	B_*b*_^*eq*^	2040.5	2029.6	2033.3	2039.6	2014.3
	B_*c*_^*eq*^	1342.7	1335.5	1337.7	1342.0	1325.4
	MUE		13.3	3.1	1.5	31.2
	MAX		21.7	7.2	2.8	50.0
	MUE%		0.54	0.38	0.05	1.28
	MAX%		0.55	0.41	0.07	1.29

a SE equilibrium rotational constants obtained correcting the experimental ground-state rotational constants by computed vibrational corrections. Experimental data are from ref (4) for uracil and ref (22) for cytosine. Computed vibrational corrections at the B3 level are from ref (76) for uracil and this work (see Table 3) for cytosine.

**Figure 1:**
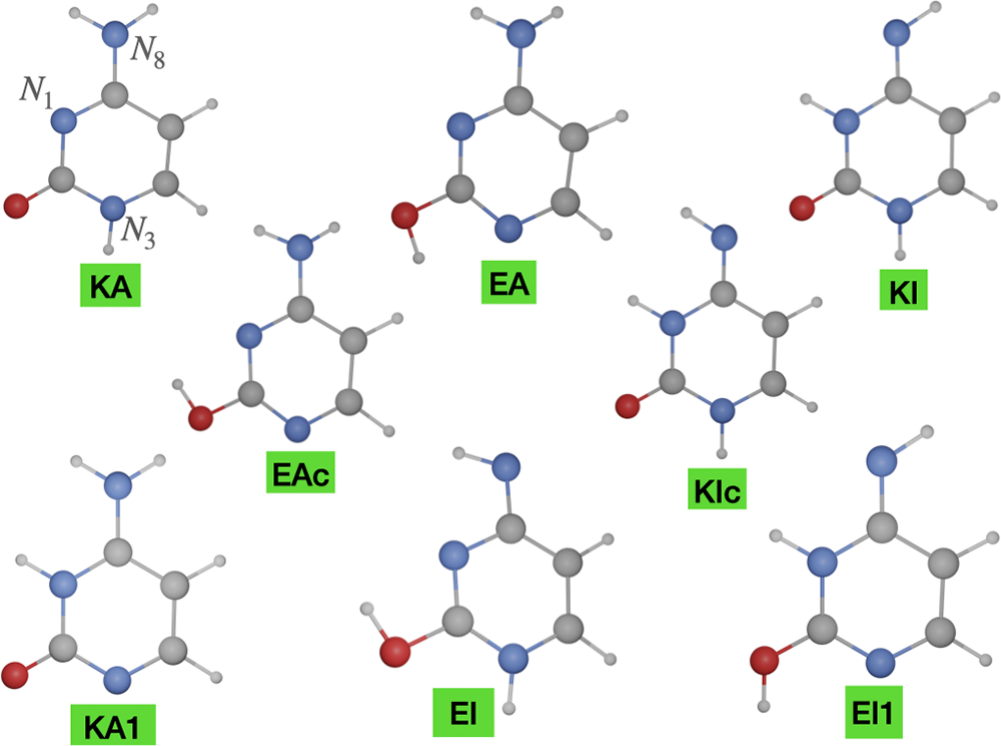
Structure of cytosine tautomers.

The number of possible tautomers (*N*_T_) of a given species is *N*_T_ = *A*!/*B*!(*A* – *B*)!, where *A* is the number of tautomeric sites and *B* is the number of labile protons. Cytosine has two endo (N1 and N3) and two exo (O7 and N8) tautomeric sites and two labile protons, so that *A* = 4, *B* = 2, and *N*_T_ = 6 (see Figure 1). These tautomers can be classified as keto-amino (KA and KA1), enol-amino (EA), keto-imino (KI), and enol-imino (EI and EI1). Furthermore, each enol and imino group shows, together with the most stable form, an additional rotamer: as a consequence, two rotamers are possible for enol-amino (labeled EA and EAc) and keto-imino (labeled KI and KIc) tautomers, whereas four rotamers are possible for each enol-imino tautomer. However, in the latter case, only the most stable rotamer (EI and EI1) will be considered in the following since it is already much less stable than the other tautomers. While all these eight species will be analyzed, the focus of the discussion will be on the five most stable ones (namely KA, EA, EAc, KI, and KIc), the only ones having non-negligible populations in the gas phase according to all the theoretical and experimental studies performed till now.

The optimized geometries collected in Tables S2–S6 of the SI show that the amino tautomers are slightly non-planar (C_1_ symmetry), whereas their imino counterparts are planar (C_*s*_ symmetry). The barriers to planarity are always very small so that effectively planar structures are expected for the ground vibrational states. However, all single point energy refinements have been performed at the non-planar geometries optimized at the PCS level.

The equilibrium rotational constants computed at different levels are compared in Tables 1 and 2 to their SE counterparts obtained from the experimental ground state values reported in ref (22) and the B3 vibrational corrections given in Table 3.

**Table 2:** Equilibrium Rotational Constants of KI and KIc Energy Minima of Cytosine Obtained by Different Methods

	parameter	SE^a^	rDSD	rDSD+CV^b^	CC-F12/j3	CC-F12/j3+CV^*b*^
KI	B_*a*_^*eq*^	3877.6	3867.7	3881.8	3863.0	3877.7
	B_*b*_^*eq*^	2037.3	2030.7	2036.2	2030.2	2035.2
	B_*c*_^*eq*^	1335.7	1331.6	1335.6	1330.8	1334.7
	MUE		6.9	1.8	8.9	1.1
	MAX		9.9	4.2	14.6	2.1
	MUE%		0.30	0.06	0.37	0.07
	MAX%		0.32	0.11	0.38	0.10
KIc	B_*a*_^*eq*^	3889.7	3880.3	3892.7	3875.2	3887.7
	B_*b*_^*eq*^	2022.8	2016.3	2023.2	2016.0	2023.0
	B_*c*_^*eq*^	1330.9	1326.8	1331.3	1326.1	1330.6
	MUE		6.7	1.3	8.7	0.8
	MAX		9.4	3.0	14.5	2.0
	MUE%		0.29	0.04	0.36	0.03
	MAX%		0.32	0.07	0.37	0.05

a SE equilibrium rotational constants obtained by correcting the experimental ground state rotational constants of ref (22) with B3 vibrational corrections given in Table 3.

b CV contribution evaluated at the MP2/C3 level.

**Table 3:** Equilibrium Rotational Constants and Vibrational Corrections for the Five Most Stable Energy Minima of Cytosine^a^

label	QC level	B_*a*_^*eq*^	B_*b*_^*eq*^	B_*c*_^*eq*^	Δ*B*_*a*_^*vib*^	Δ*B*_*b*_^*vib*^	Δ*B*_*c*_^*vib*^
	B3	3935.4	1996.1	1324.9	27.6	11.0	8.6
EA	rDSD	3963.7	2011.1	1334.9	29.3	11.6	8.9
	B3	3870.1	2014.3	1325.4	23.9	12.6	8.8
EAc	rDSD	3898.4	2029.6	1335.5	25.4	13.1	9.0
	B3	3850.9	1992.1	1312.9	27.4	9.4	7.9
KA	rDSD	3884.8	2025.9	1331.9	29.1	9.9	8.1
	B3	3830.0	2010.4	1318.4	29.4	11.0	7.7
KI	rDSD	3861.7	2026.9	1329.2	30.8	11.7	8.0
	B3	3850.9	1992.1	1312.9	28.4	11.4	7.7
KIc	rDSD	3875.2	2012.3	1324.5	29.7	12.0	8.0

a All the values are in MHz.

The results show that the B3LYP functional routinely employed in the interpretation of MW spectra can provide at most qualitative trends and that at this level, the computation of vibrational corrections is not warranted. Already rDSD/j3 computations perform a remarkable job, and extension of the basis set together with inclusion of CV correlation (which plays a comparable role) leads to remarkable quantitative agreement with the SE reference values. As a matter of fact, the relative mean unsigned error (MUE%) is always close to 0.05% and the corresponding relative maximum unsigned error (MAX%) close to 0.1%, with these values being on par with the results delivered by the most sophisticate (and much more expensive) wave-function composite methods.^55,57^ It is also remarkable that the errors are comparable for different tautomeric species, irrespective of their planar or non-planar arrangement.

In order to further investigate this aspect, the geometry of the planar KI and KIc species has been optimized also at the CCSD(T)-F12/j3 level (hereafter CC-F12/j3, vide infra for definition). The results collected in Table 2 show that the errors are larger than those delivered by our best computations, and only after inclusion of CV correlation, they come close to their PCS counterparts.

Since the errors on equilibrium rotational constants approach the expected errors of vibrational corrections computed at the B3 level, the vibrational corrections of the rotational constants have been recomputed at the rDSD/j3 level for the five most stable species (see Table 3). Noted is that that these computations are very expensive since each of them requires 67 Hessian evaluations. As a consequence, we are implementing an alternative procedure for evaluating these contributions (which require only semi-diagonal third derivatives) in a more effective way when fourth derivatives are not needed. In any case, B3 rotational constants are in semi-quantitative agreement with their rDSD/j3 counterparts, with the relative maximum unsigned error being about 5%, which corresponds to an error on ground-state rotational constants within 0.05%, i.e., slightly lower than the relative maximum error of the PCS results.

On top of accurate geometries, reliable electronic energies can be computed by methods rooted in the coupled cluster (CC) ansatz^77^ including single, double, and perturbative estimate of triple excitations (CCSD(T)).^78^ Based on this premise, the starting point of the family of “cheap” schemes (ChS) developed in the last years^5,30,33,34^ is a frozen core CCSD(T) computation in conjunction with a triple-zeta basis set.^45,49,79^ Then, in analogy with the correlation consistent composite approach (ccCA),^80,81^ CBS extrapolation and CV correlation can be taken into account with good accuracy and negligible additional cost by means of second-order Møller–Plesset perturbation theory (MP2).^82^ Replacement of conventional methods by their explicitly correlated (F12) counterparts^83,84^ further improves the reliability of “cheap” models, thanks to the strong reduction of CBS extrapolation without excessive increase of computational cost.^35,41,43^ For the same reasons explained above for geometries, the cc-pVnZ-F12 family of basis sets has been employed also for energies. Since previous studies showed that the cc-pVDZ-F12 basis set produces unsatisfactory results for third-row atoms^85^ and non-covalent interactions,^35^ the starting point is a frozen core CCSD(T)-F12 computation in conjunction with the same 3F12 basis set employed in rDSD geometry optimizations. Next, the CBS extrapolation is performed by the standard *n*^–3^ two-point formula^86^ employing MP2-F12/cc-pVnZ-F12 energies with N = T and Q (hereafter 4F12), whereas the CV contribution is incorporated as the difference between *ae* and *fc* MP2-F12 calculations, both with the same cc-pCVTZ-F12 basis set (hereafter C3F12).^87^ This recipe is summarized by the following equations:
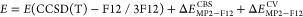
3where
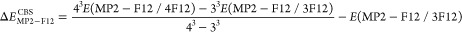
4and
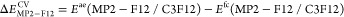
5

The *fc* approximation is employed in eqs 3 and 4, so that, the computational effort and expected accuracy of the proposed method are roughly comparable with those of the W1-F12 model employed in the study of guanine tautomers mentioned in Section 1.^10^

The relative electronic energies of all the tautomers and rotamers computed at different levels are reported in Table 4. It is quite apparent that the CV contribution cannot be neglected for accurate computations, whereas the role of CBS extrapolation becomes marginal when employing explicitly correlated methods even for a target accuracy of 10 cm^–1^ (about 0.1 kJ mol^–1^). For instance, the contribution of the CBS extrapolation to the relative stability of the KA tautomer is reduced from more than 100 to less than 10 cm^–1^ when going from conventional CCSD(T)/j3 computations to their CCSD(T)-F12/3F12 counterparts (see Table 4) without any huge increase of the required computational resources. Since the accuracy of the CBS extrapolation is limited by its evaluation at the MP2 (or MP2-F12) level, its strong reduction increases the robustness of the additive approach underlying any composite method. As already mentioned, rDSD/j3 optimized geometries are sufficiently reliable for the computation of electronic energies: as a matter of fact, the maximum difference between CC/j3 relative energies at PCS and rDSD/j3 geometries amounts to 2.1 cm^–1^ (cfr. fifth column and note (d) of Table 4). Taking the “canonical” KA species as a reference, irrespective of the employed basis set, the rDSD functional underestimates the relative stability of the EA species (105 vs a best value of −250 cm^–1^), whereas it reproduces correctly that of the KI species (295 vs a best value of 254 cm^–1^). Just the opposite behavior is observed at the MP2 level (−589 and 515 cm^–1^ for EA and KI, respectively). Finally, the energy differences between different rotamers are well reproduced by both rDSD and MP2 computations for both the EA (258 and 249 cm^–1^, respectively, to be compared to a best value of 250 cm^–1^) and the KI (615 and 608 cm^–1^, respectively, to be compared to a best value of 606 cm^–1^) tautomers. The stability of the three less stable tautomers (KA1, EI1, and EI) with respect to the EA species ranges between 2900 and 5900 cm^–1^ according to the best computations, with rDSD performing a remarkable job for EI1 and EI, whereas the over-stabilization of KA1 is comparable to that of KA.

**Table 4:** Relative Electronic Energies of All the Tautomers and Rotamers of Cytosine Computed by Different Methods and Relative rDSD/j3 Zero Point Energies (ΔZPE)^a^

tautomer	B3^b^	rDSD^c^	MP2/j3^c^	CC/j3^d^^,^^e^	ΔCBS^d^^,^^f^	CC-F12/3F12^d^	ΔCBS^d^^,^^g^	ΔCV^d^^,^^h^	total^i^	ΔZPE
EA	0.0	0.0	0.0	0.0	0.0	0.0	0.0	0.0	0.0	0.0
EAc	277.7	257.9	249.3	239.6	6.6	246.1	1.2	2.9	250.2	-5.2
KA	-601.3	-104.8	588.9	402.5	-102.7	320.0	-8.5	-23.9	287.6	-45.0
KI	82.4	185.7	1104.1	601.4	-68.4	565.4	-7.2	-16.0	542.2	101.5
KIc	741.7	800.5	1712.9	1180.3	-50.1	1160.0	-6.2	-5.7	1148.1	57.3
KA1	1809.0	2447.3	3157.9	2927.7	5.3	2898.2	0.6	0.6	2899.4	
EI1	4143.3	4271.9	4792.6	4298.5	-2.5	4330.5	-0.3	21.4	4351.6	
EI	6480.9	5880.1	6413.3	5824.6	-2.3	5867.8	-0.2	13.8	5881.4	

a All the values are in cm^–1^ (1 kJ/mol = 83.59 cm^–1^).

b At B3 geometry.

c At rDSD/j3 geometry.

d At PCS geometry.

e The corresponding values at rDSD/j3 geometry are: 0.0, 239.3, 404.6, 601.0, 1181.9, 2928.4, 4296.7, and 5825.3 cm^–1^.

f From MP2/j3 and MP2/j4 energies.

g From MP2-F12/3F12 and MP2-F12/4F12 energies.

h From ae and fc MP2-F12/C3F12 energies.

i Sum of columns 7, 8, and 9.

The relative free energies at room temperature obtained employing rDSD/j3 vibrational frequencies are given in Table 5. It is quite apparent that the main effect of ZPE (see Table 4) and TCs is to increase the relative stability of the KA species with respect to all the other tautomers (and rotamers). The same harmonic frequencies and rDSD/j3 quadrupole coupling constants are next employed, in conjunction with PCS equilibrium rotational constants and B3 anharmonic contributions, to obtain spectroscopic parameters to be compared with their experimental counterparts.

**Table 5:** Ground-State Rotational Constants (*B*_a_^0^, *B*_b_^0^, and *B*_c_^0^ in MHz) Computed with PCS Geometries and rDSD/j3 Vibrational Corrections, Together with ^14^N-Nuclear Quadrupole Coupling Constants (χ in MHz) and Electric Dipole Moment Components (μ in debye) Computed at the rDSD/j3 Level of the Five Most Stable Tautomers of Cytosine^a^

conformer	EA	EAc	KA	KI	KIc
computed					
*B*_a_^0^	3955.4	3891.9	3875.2	3851.0	3863.0
*B*_b_^0^	2009.3	2026.5	2026.0	2024.5	2011.2
*B*_c_^0^	1332.6	1333.0	1330.4	1327.6	1323.3
χ_aa_N1	-2.6818	-2.8574	1.6092	1.8615	2.1259
χ_bb_N1	1.1516	1.0054	1.4602	2.0432	1.7903
χ_cc_N1	1.5301	1.8520	-3.0694	-3.9047	-3.9162
χ_aa_N3	2.2536	2.2343	2.5229	2.1311	1.8548
χ_bb_N3	-3.7073	-3.4322	-3.5884	1.6010	2.0767
χ_cc_N3	1.4537	1.1980	1.0654	-3.7321	-3.9315
χ_aa_N8	2.1936	2.1865	2.1923	1.8367	-2.1965
χ_bb_N8	1.9946	2.0231	1.9159	-3.1411	0.9632
χ_cc_N8	-4.1882	-4.2096	-4.1082	1.3044	1.2333
μ_a_	-2.9632	-4.4160	-4.4651	-0.6290	0.6886
μ_b_	1.3316	-0.9458	4.6748	4.6635	2.3435
μ_c_	-0.6243	-0.6612	-0.4753	0.0000	0.0000
ΔG^0^	0	247	161	579	1136
experimental^b^					
*B*_a_^0^	3951.85325(32)	3889.46510(38)	3871.54618(31)	3848.18174(41)	3861.2966(12)
*B*_b_^0^	2008.95802(12)	2026.31804(12)	2024.97804(11)	2026.31068(31)	2011.41032(62)
*B*_c_^0^	1332.47228(08)	1332.86951(10)	1330.33627(08)	1327.99167(10)	1323.19999(22)
χ_aa_N1	-2.6373(13)	-2.8007(18)	1.6211(19)	1.8518(69)	1.898(23)
χ_bb_N1	1.1672(28)	1.0340(27)	1.4772(34)	2.0545(40)	2.104(28)
χ_cc_N1	1.4701(28)	1.7667(27)	-3.0983(34)	-3.9063(40)	-4.002(28)
χ_aa_N3	2.2619(20)	2.2371(23)	2.5217(12)	2.1383(33)	2.105(20)
χ_bb_N3	-3.6570(22)	-3.3890(25)	-3.5140(16)	1.6064(42)	1.764(28)
χ_cc_N3	1.3951(22)	1.1519(25)	0.9923(16)	-3.7448(42)	-3.870(28)
χ_aa_N8	2.2167(17)	2.2237(17)	2.1802(17)	1.8033(87)	-2.091(15)
χ_bb_N8	1.9511(20)	1.9832(20)	1.8429(26)	-3.1572(58)	0.940(14)
χ_cc_N8	-4.1678(20)	-4.2069(20)	-4.0231(26)	1.3539(58)	1.151(14)
ΔG^0^	0	165	40	290	/

a The computed relative free energies at room temperature (Δ*G*^0^ in cm^–1^) are also reported.

b From ref (22) with standard errors shown in parentheses in units of the last digits.

Concerning MW spectra, inspection of Table 5 shows the already mentioned quantitative agreement between theory and experiment, which is further slightly improved when using rDSD/j3 vibrational corrections in place of their B3 counterparts employed in Tables 1 and 2. For instance, the computed (experimental) differences between the rotational constants of EA and EAc rotamers are in full quantitative agreement: Δ*B*_a_ = −63.5 (−62.4) MHz, Δ*B*_b_ = 17.2 (17.4) MHz, and Δ*B*_c_ = 0.4 (0.4) MHz. The agreement is indeed one order of magnitude better than that obtained at the MP2 level by Alonso et al.,^22^ with this finding being remarkable for assignment purposes since the quadrupole coupling constants of EA and EAc are very similar for all the ^14^N nuclei. In particular, the positive values of χ_cc_ for ^14^N_1_ and ^14^N_3_ (between 1.2 and 1.9 MHz from both theory and experiment) indicate that these atoms are pyridinic nitrogen atoms. In the same vein, the negative χ_aa_ values of ^14^N_1_ (close to those observed and computed for aniline^88^ and p-toluidine^89^) indicate that this atom belongs to an amino group. The quadrupolar couplings of ^14^N_3_ and ^14^N_8_ do not change very much for the KA tautomer, whereas those of ^14^N_1_ change radically, with the value of χ_cc_ becoming close to those of uracil^4^ and thymine.^6^ Finally, the KI and KIc rotamers show ^14^N_8_ coupling constants different from those of all the other species, with this suggesting the off-cycle imino nature of this atom, despite the lack of any experimental reference for such moiety.

The relative intensity of the quadrupole component lines of the observed species (except for the too weak intensity of KIc) was used together with dipole moment components computed at the MP2 level to estimate the relative free energies of the different species.^90^ While there is full agreement between theory and experiment concerning general trends and, in particular, the larger abundance of the EA species in the gas phase with respect to the canonical KA form, from a quantitative point of view, the differences are significant. However, it must be taken into account that the experimental estimate is based on a number of assumptions, like that under jet-cooling conditions, all the isomers/conformers are populated or depopulated in the same way by collisions. From that point of view, matrix isolation studies have the advantage that due to the fast freezing process, the gas phase populations of different species are conserved in the low-temperature matrix in the presence of sufficiently high (roughly > 400 cm^–1^) energy barriers governing the inter-conversion between different structures.^91,92^ Thus, if thermal equilibrium is established in the gas phase, then the abundances observed in the matrix can be compared directly to QC ratios computed at the evaporation temperature of the sample. The most accurate experimental values have been obtained from the ratios of several IR bands^28^ and correspond to relative populations of 1.00, 0.59, 0.50, and 0.18 at 450 K for EA, EAc, KA, and KI tautomers, respectively. The agreement with the corresponding computed values (1.00, 0.45, 0.60, and 0.16) is indeed remarkable. Comparison with the results obtained using computed electronic energies in place of free energies (1.0, 0.45, 0.40, and 0.18) shows that the main effect of ZPE and thermal contributions is to stabilize the KA tautomer, leading to a slightly larger population of KA with respect to EAc.

In any case, the population of the KI tautomer is not negligible, thus confirming the revision proposed in ref (28) of the very small values estimated in refs (15, 93) It is also noteworthy that, again in agreement with the experimental estimates of ref (28) in the gas phase, the combined mole fraction of the enol-amine forms (EA+EAc) at 450 K is very large (0.66 from computations and 0.70 from experiment), especially when taking into account that only the KA tautomer has been detected in the solid state or in aqueous solution.

Coming to vibrational spectra, the computed harmonic and anharmonic (VPT2) frequencies and intensities are compared to their experimental counterparts recorded in inert matrices for EA (Table 6) and KA (Table 7) tautomers. Since experimental data are not available for the KI tautomer, only the computed values are given in Table 8. In general terms, for most modes, the differences between calculated VPT2 frequencies and their experimental counterparts are of the order of a few wave-numbers without any need of scaling parameters. Larger differences are observed for the C=O stretching of the KA tautomer (overestimated by about 40 cm^–1^). Since the assignment parallels those of refs (15, 94) only a short summary of the most significant features is given in the following.

**Table 6:** Calculated and Experimental IR Spectra of the EA Tautomer of Cytosine

mode^a^	B3/h	B3/anh	rDSD/h	rDSD/B3^b^	exp.^c^
1 ν(OH)	3715	3525	3793	3603(99)	3592(168)
2 ν(asNH2)	3709	3560	3739	3590(46)	3564(98)
3 ν(sNH2)	3588	3450	3608	3470(71)	3446(154)
4 ν(C5H)	3217	3090	3223	3096(5)	
5 ν(C6H)	3184	3062	3183	3063(14)	
6 ν(C5C6)	1674	1625	1671	1623(565)	1622(645)
7 β(scNH2)	1651	1607	1643	1599(1 2)	1589(64)
8 ν(N3C4)	1616	1570	1625	1579(256)	1570(52)
9 β(C5H)	1531	1494	1534	1497(36)	1496(73)
10 ν(CO)	1476	1440	1478	1442(408)	1427(285)
11 ν(C5C6)	1408	1383	1410	1385(46)	
12 β(C6H)	1357	1317	1353	1313(173)	1320(72)
13 β(OH)	1311	1279	1291	1259(27)	
14 ν(N1C2)	1250	1216	1250	1216(34)	1196(124)
15 β(C5H)	1137	1115	1133	1111(39)	1110(29)
16 β(roNH2)	1106	1067	1106	1067(43)	1083(60)
17 γ(C6H)	1008	989	1008	989(9)	
18 β(R1)	995	973	1006	984(1)	980(30)
19 ν(C4C5)	992	973	995	976(16)	955(3)
20 γ(CO)	816	810	827	821(51)	807(69)
21 γ(C5H)	796	784	804	792(1)	
22 β(R1)	792	778	796	782(7)	781(30)
23 γ(CO)	716	716	728	728(4)	710(8)
24 β(R3)	601	593	601	593(1)	
25 τ(OH)	563	550	562	551(28)	557(21)
26 β(R2)	571	522	562	523(71)	520(221)
27 τ(toNH2)	511	489	511	489(15)	507(67)
28 τ(toNH2)	491	460	493	462(12)	498(42)
29 τ(R3)	450	444	451	445(16)	443(13)
30 β(CN8)	343	329	342	328(45)	343(11)
31 τ(invNH2)	321	-216	348	348(219)	235(244)
32 τ(invNH2)	220	216	220	216(12)	
33 τ(R2)	186	185	187	186(1)	

a Notation for modes: ν, stretching (as, asymmetric; s, symmetric); β, bending or ring deformation; ro, rocking; sc, scissoring; γ, out-of-plane-bending; τ, out-of-plane ring deformation; to, torsional; inv: inversion.

b rDSD/j3 harmonic frequencies with B3 anharmonic corrections (column 3 - column 2) and (in parenthesis) rDSD/j3 harmonic intensities.

c In Ar matrix from ref (94).

**Table 7:** Calculated and Experimental IR Spectra of the KA Tautomer of Cytosine

mode^a^	B3/h	B3/anh	rDSD/h	rDSD/B3^b^	exp.^c^
1 ν(asNH2)	3715	3577	3749	3611(50)	3564(122)
2 ν(N1H)	3612	3441	3640	3469(82)	3472(123)
3 ν(sNH2)	3590	3459	3611	3480(88)	3441(192)
4 ν(C5H)	3236	3099	3243	3106(1)	
5 ν(C6H)	3215	3104	3216	3105(2)	
6 ν(C2O)	1776	1746	1774	1744(751)	1719(782)
7 ν(C5C6)	1696	1654	1704	1662(470)	1656(384)
8 β(scNH2)	1657	1609	1643	1595(130)	1598(312)
9 ν(N3C4)	1575	1535	1586	1546(162)	1539(113)
10 ν(N3C4)	1513	1488	1516	1491(154)	1475(242)
11 β(N1H)	1453	1419	1453	1419(73)	1423(30)
12 ν(C4N)	1364	1345	1365	1346(59)	1337(66)
13 ν(C2N3)	1265	1237	1272	1244(22)	1244(28)
14 β(C6H)	1228	1205	1221	1198(55)	1196(49)
15 β(C5H)	1134	1118	1130	1114(1)	
16 β(roNH2)	1099	1043	1108	1052(44)	1090(56)
17 β(R1)	990	970	992	972(1)	
18 γ(C6H)	959	942	975	958(1)	
19 ν(N1C2)	929	901	937	909(3)	
20 γ(C2O)	773	766	792	775(40)	781(32)
21 γ(C2O)	768	762	779	773(7)	
22 γ(C4N)	763	758	773	768(6)	747(34)
23 γ(C5H)	725	715	734	724(31)	716(39)
24 γ(N1H)	628	621	630	623(59)	614(85)
25 β(R2)	578	572	576	570(2)	575(76)
26 β(R3)	547	545	546	544(5)	568(58)
27 τ(toNH2)	532	541	536	545(3)	535(24)
28 β(C2O)	522	566	529	573(14)	
29 τ(R2)	397	398	401	402(19)	400(23)
30 β(C4N)	359	353	359	353(2)	
31 τ(invNH2)	246	-710	266	266(233)	235(244)
32 τ(R3)	202	189	204	191(6)	
33 τ(R2)	135	136	133	134(2)	

a Notation for modes: ν, stretching (as, asymmetric; s, symmetric); β, bending or ring deformation; ro, rocking; sc, scissoring; γ, out-of-plane-bending; τ, out-of-plane ring deformation; to, torsional; inv: inversion.

b rDSD/j3 harmonic frequencies with B3 anharmonic corrections (column 3 - column 2) and (in parenthesis) rDSD/j3 harmonic intensities.

c In Ar matrix from ref (94).

**Table 8:** Calculated IR Spectra of the KI Tautomer of Cytosine

mode^a^	B3/h	B3/anh	rDSD/h	rDSD/B3^b^
1 ν(N1H)	3640	3467	3668	3495(114)
2 ν(N3H)	3597	3423	3618	3444(65)
3 ν(N8H)	3493	3314	3523	3344(11)
4 ν(C5H)	3245	3108	3248	3111(1)
5 ν(C6H)	3224	3084	3222	3082(2)
6 ν(C2O)	1803	1765	1803	1765(814)
7 ν(C5C6)	1721	1682	1725	1686(421)
8 β(N8H)	1663	1620	1659	1616(23)
9 ν(N3C4)	1509	1475	1514	1480(95)
10 ν(N3C4)	1445	1411	1453	1419(78)
11 β(N1H)	1424	1384	1417	1377(11)
12 ν(C4N)	1407	1372	1409	1374(21)
13 ν(C2N3)	1311	1270	1323	1282(19)
14 β(C6H)	1227	1210	1219	1202(92)
15 β(C5H)	1152	1119	1155	1122(140)
16 β(N8H)	1097	1078	1097	1078(1)
17 β(R1)	993	976	998	981(7)
18 β(C6H)	970	949	974	953(9)
19 ν(N1C2)	770	750	772	752(5)
20 β(R2)	567	560	566	559(7)
21 β(R3)	534	528	536	530(3)
22 β(CO)	518	513	515	510(32)
23 β(C4N)	376	376	376	376(10)
24 γ(CO)	953	935	966	948(1)
25 γ(C4N)	828	808	841	821(82)
26 γ(CO)	769	757	779	767(4)
27 γ(C5H)	736	741	758	763(36)
28 γ(N1H)	699	689	708	698(10)
29 τ(NH)	657	646	659	648(95)
30 τ(R2)	527	529	518	520(35)
31 τ(NH)	386	383	383	380(21)
32 τ(R3)	157	159	152	154(2)
33 τ(R2)	137	139	130	132(0)

a Notation for modes: ν, stretching (as, asymmetric; s, symmetric); β, bending or ring deformation; ro, rocking; sc, scissoring; γ, out-of-plane-bending; τ, out-of-plane ring deformation; to, torsional; inv: inversion.

b rDSD/j3 harmonic frequencies with B3 anharmonic corrections (column 3 - column 2) and (in parenthesis) rDSD/j3 harmonic intensities.

The bands observed in the high-frequency region (3700–3400 cm^–1^) originate from the stretching vibrations of the OH, NH_2_, and NH groups, whose large anharmonic contributions are well captured by the VPT2 approach. The assignment of these bands agrees with previous ones and, in particular, the OH band computed at 3603 cm^–1^ and experimentally observed at 3592 cm^–1^ for the EA tautomer disappears in both ketonic forms (KA and KI).

In the mean frequency region (1700–700 cm^–1^), most bands are due to the ring stretching vibrations mixed with CH bendings. Those bands are usually well predicted even at the harmonic level, and their assignment is quite straightforward. Furthermore, the assignment of the carbonyl group stretching, which is the most intense band in the spectrum of KA and KI tautomers, is obvious.

The more problematic assignment of the bands due to the NH_2_ scissoring in EA and KA tautomers is also quite straightforward from the reported computations, and the same applies to the sequence of the strong ring stretching band and the weaker NH_2_ scissoring band in the EA tautomer. The typical splitting of vibrations with significant contributions from βOH modes of heterocyclic compounds^95^ is also well recognized. The splitting of the experimental band of CO(H) stretching at 1427 cm^–1^ (together with that of other weaker bands) is diagnostic of the presence of both EA and EAc conformers in the matrix. It is remarkable that this feature is reproduced by the computations since the EA band at 1442 cm^–1^ (Table 6) is shifted to 1456 cm^–1^ for EAc (data not shown). Contradictory assignments were proposed for the band observed at 1749 cm^–1^ for the KA tautomer: VPT2 computations show unequivocally that it is due to a Fermi resonance between the C=O stretching and a combination of lower frequency modes. A similar Fermi resonance has been experimentally observed and reproduced by VPT2 computations in the case of uracil.^3,96^ A weak band near 1750 cm^–1^ observed for the EA tautomer can be confidently assigned to a combination vibration. At the same time, no absorption is present in the spectrum of the EA tautomer near 1670 cm^–1^ so that a rather weak band at 1589 cm^–1^ (computed at 1599 cm^–1^) can be confidently assigned to NH_2_ scissoring.

The low-frequency range (below 700 cm^–1^) is populated by bands due to both in plane and out of plane vibrations. The former type of vibrations originates from bendings involving the ring, together with the CO and CN8 groups, which are well predicted already at the harmonic level and do not present specific assignment problems. Also more subtle effects, like the couplings between in plane vibrations and twisting vibrations of amino-groups, are well evidenced. Finally, the frequencies of the wagging modes of the CO and CH groups are well predicted, and this is also the case for the out-of-plane ring deformations. As expected, the VPT2 model is not able to reproduce the anharmonicity of NH_2_ inversion (mode 31 of EA and KA), whose shape requires more refined models. On the other hand, all the other low-frequency modes (including OH torsion in the EA tautomer) do not present particular problems.

In summary, the VPT2 model based on rDSD/B3 hybrid force fields confirms its robustness and reliability without the need of any empirical parameter.^54,68^

## 4 Concluding Remarks

In this paper, a general strategy aimed at the unbiased disentanglement of the conformational behaviour of biomolecule building blocks has been extended to tautomeric equilibria. Accurate structures and relative energies are obtained by integrating DFT and wave-function composite schemes. Next, the spectroscopic parameters of sufficiently populated tautomers (and, possibly, rotamers) can be safely computed by modern double-hybrid functionals. The results obtained for cytosine are in full agreement with the available spectroscopic data and permit their unbiased interpretation in terms of the cooperation or competition between different stereo-electronic effects.

In more general terms, the results of the present investigation confirm that highly reliable analysis of structural and spectroscopic features is today possible for biomolecule building blocks even in the presence of subtle tautomeric equilibria by methods coupling accuracy and feasibility without the need of any empirical parameter.

## Supporting Information Available

Sample input for a PCS computation and optimized geometries of uracil (Table S1) and of the five most stable cytosine tautomers and rotamers (Tables S2 to S6) (PDF)
