# Supplementary material for: DFT Meets Wave-Function Composite Methods for Characterizing Cytosine Tautomers in the Gas Phase
Source: J Chem Theory Comput. 2023 Jul 21;19(15):4970–81. doi: 10.1021/acs.jctc.3c00465 (PMC10413851; doi:10.1021/acs.jctc.3c00465)
Supplement: Supplementary file 1 — ct3c00465_si_001.pdf [file ct3c00465_si_001.pdf]

Supporting Information: DFT Meets Wave-Function Composite Methods for Characterizing Cytosine Tautomers in the Gas Phase

Vincenzo Barone*

Scuola Normale Superiore di Pisa, piazza dei Cavalieri 7, 56126 Pisa, Italy

E-mail: vincenzo.barone@sns.it

The following input stream for G16 generates three geometries. The optimized Z-matrix parameters need be combined (rDSD+MP2-ae-MP2-fc) and the resulting set defines the PCS geometry.

```
--link1--
%mem=40GB
%nprocshared=8
%chk=ura.chk
#dsdpbep86 gen fopt=zmatrix output=pickett
empiricaldispersion=gd3bj
iop(3/125=0079905785,3/78=0429604296,3/76=0310006900,3/74=1004,
3/174=0437700,3/175=-1,3/176=0,3/177=1,3/178=5500000)

uracil rDSD/3F12

 0 1
 N
 C,1,CN
 N,2,NC,1,NCN
 C,3,C1N,2,C1NC,1,T0,0
 C,4,CC,3,CCN,2,T0,0
 C,5,C2C,4,C2CC,3,T0,0
 H,1,HN,2,HNC,3,T180,0
 O,2,OC,1,OCN,7,T0,0
 H,3,H1N,2,H1NC,1,T180,0
 H,4,HC,3,HCN,2,T180,0
 H,5,H1C,4,H1CC,3,T180,0
 O,6,O1C,5,O1CC,4,T180,0

 CN=1.3784
 NC=1.3844
 NCN=113.2060
 C1N=1.3719
 C1NC=123.4525
 CC=1.3450
 CCN=121.9858
 C2C=1.4560
 C2CC=119.6272
 HN=1.0100
 HNC=115.5853
 OC=1.2116
 OCN=123.9428
 H1N=1.0057
 H1NC=115.2844
 HC=1.0812
 HCN=115.3776
 H1C=1.0778
 H1CC=121.8821
 O1C=1.2145
 O1CC=125.9811
```

```
 T0=0.0
 T180=180.0

-H     0
S    3   1.00
     82.6400000                   0.0020060
     12.4100000                   0.0153430
      2.8240000                   0.0755790
S    1   1.00
      0.7977000                   1.0000000
S    1   1.00
      0.2581000                   1.0000000
S    1   1.00
      0.0898900                   1.0000000
P    1   1.00
      1.5513000                   1.0000000
P    1   1.00
      0.5580000                   1.0000000
P    1   1.00
      0.2007000                   1.0000000
D    1   1.00
      0.4648000                   1.0000000
****
-C     0
S    9   1.00
  33980.0000000                   0.0000910
   5089.0000000                   0.0007040
   1157.0000000                   0.0036930
    326.6000000                   0.0153600
    106.1000000                   0.0529290
     38.1100000                   0.1470430
     14.7500000                   0.3056310
      6.0350000                   0.3993450
      2.5300000                   0.2170510
S    9   1.00
  33980.0000000                  -0.0000190
   5089.0000000                  -0.0001510
   1157.0000000                  -0.0007850
    326.6000000                  -0.0033240
    106.1000000                  -0.0115120
     38.1100000                  -0.0341600
     14.7500000                  -0.0771730
      6.0350000                  -0.1414930
      2.5300000                  -0.1180190
S    1   1.00
      0.7355000                   1.0000000
S    1   1.00
      0.2905000                   1.0000000
S    1   1.00
      0.1111000                   1.0000000
S    1   1.00
      0.0414500                   1.0000000
P    2   1.00
```

```
      34.5100000                    0.0053780
       7.9150000                    0.0361320
P     1   1.00
       2.3680000                    1.0000000
P     1   1.00
       0.8132000                    1.0000000
P     1   1.00
       0.2890000                    1.0000000
P     1   1.00
       0.1007000                    1.0000000
P     1   1.00
       0.0321800                    1.0000000
D     1   1.00
       2.2805000                    1.0000000
D     1   1.00
       0.7834000                    1.0000000
D     1   1.00
       0.2691000                    1.0000000
F     1   1.00
       1.0371000                    1.0000000
F     1   1.00
       0.3459000                    1.0000000
****
-N     0
S     9   1.00
   45840.0000000                    0.0000920
    6868.0000000                    0.0007170
    1563.0000000                    0.0037490
     442.4000000                    0.0155320
     144.3000000                    0.0531460
      52.1800000                    0.1467870
      20.3400000                    0.3046630
       8.3810000                    0.3976840
       3.5290000                    0.2176410
S     9   1.00
   45840.0000000                   -0.0000200
    6868.0000000                   -0.0001590
    1563.0000000                   -0.0008240
     442.4000000                   -0.0034780
     144.3000000                   -0.0119660
      52.1800000                   -0.0353880
      20.3400000                   -0.0800770
       8.3810000                   -0.1467220
       3.5290000                   -0.1163600
S     1   1.00
       1.0540000                    1.0000000
S     1   1.00
       0.4118000                    1.0000000
S     1   1.00
       0.1552000                    1.0000000
S     1   1.00
       0.0546400                    1.0000000
P     2   1.00
```

```
     49.3300000                  0.0055330
     11.3700000                  0.0379620
P    1   1.00
      3.4350000                  1.0000000
P    1   1.00
      1.1820000                  1.0000000
P    1   1.00
      0.4173000                  1.0000000
P    1   1.00
      0.1428000                  1.0000000
P    1   1.00
      0.0440200                  1.0000000
D    1   1.00
      3.0521000                  1.0000000
D    1   1.00
      1.0393000                  1.0000000
D    1   1.00
      0.3539000                  1.0000000
F    1   1.00
      1.3498000                  1.0000000
F    1   1.00
      0.4638000                  1.0000000
****
-O     0
S    9   1.00
  61420.0000000                  0.0000900
   9199.0000000                  0.0006980
   2091.0000000                  0.0036640
    590.9000000                  0.0152180
    192.3000000                  0.0524230
     69.3200000                  0.1459210
     26.9700000                  0.3052580
     11.1000000                  0.3985080
      4.6820000                  0.2169800
S    9   1.00
  61420.0000000                 -0.0000200
   9199.0000000                 -0.0001590
   2091.0000000                 -0.0008290
    590.9000000                 -0.0035080
    192.3000000                 -0.0121560
     69.3200000                 -0.0362610
     26.9700000                 -0.0829920
     11.1000000                 -0.1520900
      4.6820000                 -0.1153310
S    1   1.00
      1.4280000                  1.0000000
S    1   1.00
      0.5547000                  1.0000000
S    1   1.00
      0.2067000                  1.0000000
S    1   1.00
      0.0695900                  1.0000000
P    2   1.00
```

```
     63.4200000              0.0060440
     14.6600000              0.0417990
P    1   1.00
      4.4590000              1.0000000
P    1   1.00
      1.5310000              1.0000000
P    1   1.00
      0.5302000              1.0000000
P    1   1.00
      0.1750000              1.0000000
P    1   1.00
      0.0534800              1.0000000
D    1   1.00
      3.3422000              1.0000000
D    1   1.00
      1.1491000              1.0000000
D    1   1.00
      0.3951000              1.0000000
F    1   1.00
      1.4956000              1.0000000
F    1   1.00
      0.5379000              1.0000000
****


--link1--
%mem=40GB
%nprocshared=8
%chk=ura.chk
#MP2=fc gen geom=checkpoint fopt=zmatrix

uracil MP2-fc/C3

 0 1

-H 0
 cc-pVTZ
 ****
-C    0
 cc-pVTZ
S    1   1.00
     11.8760000              1.0000000
S    1   1.00
      4.2920000              1.0000000
P    1   1.00
     33.1900000              1.0000000
P    1   1.00
      8.7780000              1.0000000
D    1   1.00
     14.8390000              1.0000000
****
 -N    0
 cc-pVTZ
```

```
S    1   1.00
     16.2010000              1.0000000
S    1   1.00
      5.9520000              1.0000000
P    1   1.00
     44.8490000              1.0000000
P    1   1.00
     11.8710000              1.0000000
D    1   1.00
     14.2000000              1.0000000
****
-O     0
 cc-pVTZ
S    1   1.00
     21.0320000              1.0000000
S    1   1.00
      7.8450000              1.0000000
P    1   1.00
     57.4370000              1.0000000
P    1   1.00
     15.1590000              1.0000000
D    1   1.00
     15.8580000              1.0000000
****


--link1--
%mem=40GB
%nprocshared=8
%chk=ura.chk
#MP2=full chkbas geom=checkpoint fopt=zmatrix

uracil MP2-ae/C3

 0 1
```

Table S1: Uracil

```
N
C,1,CN
N,2,NC,1,NCN
C,3,C1N,2,C1NC,1,T0,0
C,4,CC,3,CCN,2,T0,0
C,5,C2C,4,C2CC,3,T0,0
H,1,HN,2,HNC,3,T180,0
O,2,OC,1,OCN,7,T0,0
H,3,H1N,2,H1NC,1,T180,0
H,4,HC,3,HCN,2,T180,0
H,5,H1C,4,H1CC,3,T180,0
O,6,O1C,5,O1CC,4,T180,0

T0=0.0 and T180=180 fixed
```

Param.	rDSD/j3	rDSD	MP2/C3 fc	MP2 C3 ae	rDSD+CV	SE
CN	1.3800	1.3788	1.3793	1.3768	1.3763	1.3763
NC	1.3859	1.3847	1.3845	1.3822	1.3824	1.3817
NCN	113.1801	113.1977	112.7169	112.7362	113.2170	113.29
C1N	1.3734	1.3723	1.3698	1.3674	1.3699	1.3712
C1NC	123.4601	123.4590	123.6418	123.6098	123-4270	123.47
CC	1.3468	1.3454	1.3469	1.3442	1.3427	1.3440
CCN	121.9815	121.9850	121.9629	121.9886	122.0123	121.92
C2C	1.4572	1.4565	1.4534	1.4503	1.4534	1.4555
C2CC	119.6330	119.6251	119.7681	119.7581	119.6151	119.55
HN	1.0110	1.0107	1.0105	1.0096	1.0098	1.0090
HNC	115.5728	115.5824	115.3023	115.3313	115.6114	115.58
OC	1.2142	1.2121	1.2140	1.2122	1.2103	1.2097
OCN	123.9539	123.9477	124.1937	124.1891	123.9431	123.86
H1N	1.0067	1.0065	1.0058	1.0049	1.0056	1.0046
H1NC	115.2891	115.2711	115.1230	115.1388	115.2869	115.17
HC	1.0820	1.0821	1.0798	1.0786	1.0809	1.0793
HCN	115.3887	115.3781	115.4862	115.5075	115.3568	115.33
H1C	1.0785	1.0787	1.0763	1.0751	1.0775	1.0766
H1CC	121.8678	121.8845	121.6976	121.5700	121.7569	122.08
O1C	1.2170	1.2149	1.2174	1.2157	1.2132	1.2124
O1CC	125.9849	125.9787	126.1399	126.1388	125.9776	125.81

Table S2: EA

```
N
C,1,CN
N,2,NC,1,NCN
C,3,C1N,2,C1NC,1,T0,0
C,4,CC,3,CCN,2,T1,0
C,5,C2C,4,C2CC,3,T2,0
O,2,OC,1,OCN,6,T180,0
H,7,H1O,2,H1OC,1,T181,0
H,4,HC,3,HCN,2,T182,0
H,5,H1C,4,H1CC,3,T183,0
N,6,N1C,5,N1CC,4,T184,0
H,11,H2N,6,H2NC,5,T3,0
H,11,H3N,6,H3NC,5,T185,0
```

Param.	rDSD/j3	rDSD	MP2/C3 fc	MP2 C3 ae	DSD+CV
CN	1.3286	1.3272	1.3279	1.3254	1.3247
NC	1.3319	1.3303	1.3301	1.3278	1.3280
NCN	128.4522	128.3705	128.4658	128.3701	128.2748
C1N	1.3434	1.3421	1.3415	1.3391	1.3394
C1NC	114.5087	114.5906	114.4917	114.5542	114.6531
CC	1.3777	1.3767	1.3771	1.3747	1.3743
CCN	123.3545	123.3255	123.1929	123.1706	123.3032
C2C	1.4083	1.4076	1.4027	1.4003	1.4052
C2CC	116.2125	116.1983	116.4244	116.4434	116.2173
OC	1.3436	1.3420	1.3453	1.3431	1.3398
OCN	114.8420	114.9023	114.7593	114.8241	114.9671
H1N	0.9666	0.9658	0.9663	0.9655	0.9650
H1NC	105.7083	105.8733	104.7593	104.8371	105.9511
HC	1.0848	1.0852	1.0827	1.0815	1.0840
HCN	116.0079	116.0111	116.1210	116.1565	116.0466
H1C	1.0813	1.0817	1.0792	1.0780	1.0805
H1CC	121.7875	121.8111	121.5928	121.5794	121.7977
N1C	1.3633	1.3619	1.3679	1.3649	1.3589
N1CC	121.8986	121.9128	121.9111	121.9299	121.9316
H2N	1.0043	1.0041	1.0039	1.0027	1.0029
H2NC	118.9679	119.0024	117.4350	117.6861	119.2535
H3N	1.0065	1.0063	1.0061	1.0050	1.0052
H3NC	116.2167	116.2119	114.5239	114.7497	116.4377
T0	0.0986	0.1115	0.1914	0.1824	0.1025
T01	0.1284	0.1344	0.1581	0.1697	0.1460
T02	-0.1152	-0.1292	-0.2393	-0.2310	-0.1209
T180	179.8942	179.8571	179.8574	179.8576	179.8573
T181	179.8573	179.8609	179.8557	179.7996	179.8048
T182	-179.9206	-179.9179	-179.9324	-179.9132	-179.8987
T183	179.5410	179.5302	179.4252	179.4303	179.5353
T184	177.9782	178.0157	177.4587	177.5640	178.1210
T03	18.9520	18.9511	24.6657	23.8433	19.1217
T185	167.8892	168.0285	165.5107	165.8886	168.4064

Table S3: EAc

```
N
C,1,CN
N,2,NC,1,NCN
C,3,C1N,2,C1NC,1,T0,0
C,4,CC,3,CCN,2,T1,0
C,5,C2C,4,C2CC,3,T2,0
O,2,OC,1,OCN,6,T180,0
H,7,H1O,2,H1OC,1,T4,0
H,4,HC,3,HCN,2,T182,0
H,5,H1C,4,H1CC,3,T183,0
N,6,N1C,5,N1CC,4,T184,0
H,11,H2N,6,H2NC,5,T3,0
H,11,H3N,6,H3NC,5,T185,0
```

Parameter	rDSD/j3	rDSD	MP2/C3 fc	MP2 C3 ae	rDSD+CV
CN	1.3356	1.3342	1.3339	1.3315	1.3318
NC	1.3258	1.3243	1.3250	1.3227	1.3220
NCN	128.3971	128.3122	128.4005	128.3052	128.2169
C1N	1.3424	1.3410	1.3404	1.3379	1.3385
C1NC	114.0850	114.1702	114.0095	114.0792	114.2399
CC	1.3798	1.3788	1.3793	1.3768	1.3763
CCN	123.9152	123.8848	123.8071	123.7769	123.8546
C2C	1.4055	1.4047	1.3997	1.3972	1.4022
C2CC	116.2304	116.2161	116.4389	116.4655	116.2427
OC	1.3438	1.3422	1.3455	1.3434	1.3401
OCN	116.0798	116.1019	116.1526	116.1935	116.1428
H1N	0.9660	0.9652	0.9657	0.9650	0.9645
H1NC	106.1813	106.3472	105.2293	105.3201	106.4380
HC	1.0850	1.0854	1.0829	1.0817	1.0842
HCN	115.7755	115.7829	115.8559	115.8925	115.8195
H1C	1.0813	1.0817	1.0793	1.0781	1.0805
H1CC	121.8279	121.8506	121.6422	121.6205	121.8289
N1C	1.3652	1.3638	1.3697	1.3668	1.3609
N1CC	122.1510	122.1668	122.2231	122.2319	122.1756
H2N	1.0046	1.0044	1.0042	1.0031	1.0033
H2NC	118.4956	118.5343	116.9866	117.2077	118.7554
H3N	1.0064	1.0062	1.0060	1.0049	1.0051
H3NC	116.2179	116.2155	114.5439	114.7633	116.4349
T0	0.1878	0.1908	0.2583	0.2727	0.2052
T01	0.1267	0.1330	0.1705	0.1594	0.1219
T02	-0.1727	-0.1846	-0.2935	-0.2844	-0.1755
T180	179.7992	179.7854	179.7721	179.7592	179.7725
T181	-0.43234	-0.4218	-0.5323	-0.5381	-0.4276
T182	-179.9209	-179.9240	-179.9107	-179.9103	-179.9236
T183	179.5110	179.5103	179.4133	179.4307	179.5277
T184	177.9105	177.9492	177.4090	177.5285	178.0687
T03	19.6945	19.6953	24.9557	24.1626	19.9022
T185	166.7286	166.8824	164.4192	164.7239	167.1871

Table S4: KA

```
N
C,1,CN
N,2,NC,1,NCN
C,3,C1N,2,C1NC,1,T0
C,4,CC,3,CCN,2,T01
C,5,C2C,4,C2CC,3,T02
O,2,OC,1,OCN,6,T180
H,3,H1N,2,H1NC,1,T181
H,4,HC,3,HCN,2,T182
H,5,H1C,4,H1CC,3,T183
N,6,N1C,5,N1CC,4,T184
H,11,H2N,6,H2NC,5,T03
H,11,H3N,6,H3NC,5,T185
```

Parameter	rDSD/j3	rDSD	MP2/C3 fc	MP2 C3 ae	rDSD+CV
CN	1.3733	1.3723	1.3741	1.3714	1.3696
NC	1.4154	1.4144	1.4132	1.4108	1.4121
NCN	116.3866	116.3540	116.0290	116.0099	116.3349
C1N	1.3532	1.3521	1.3502	1.3480	1.3499
C1NC	123.4664	123.4687	123.8307	123.8022	123.4402
CC	1.3546	1.3535	1.3548	1.3523	1.3510
CCN	119.9403	119.9545	119.7250	119.7519	119.9814
C2C	1.4379	1.4374	1.4318	1.4295	1.4351
C2CC	115.9758	115.9661	115.9784	115.9973	115.9850
OC	1.2174	1.2151	1.2168	1.2152	1.2135
OCN	125.0646	125.0716	125.1328	125.1528	125.0916
H1N	1.0079	1.0078	1.0074	1.0065	1.0069
H1NC	115.2355	115.2072	114.9512	114.9488	115.2048
HC	1.0822	1.0825	1.0803	1.0791	1.0813
HCN	116.8533	116.8371	116.9823	116.9949	116.8497
H1C	1.0796	1.0800	1.0774	1.0762	1.0788
H1CC	121.6260	121.6443	121.4490	121.4382	121.6335
N1C	1.3590	1.3579	1.3627	1.3595	1.3547
N1CC	118.6833	118.6822	118.5252	118.5556	118.7126
H2N	1.0035	1.0034	1.0030	1.0018	1.0022
H2NC	120.1691	120.0938	118.6776	118.9847	120.4009
H3N	1.0064	1.0063	1.0059	1.0048	1.0052
H3NC	116.8393	116.7364	115.1859	115.4454	116.9959
T0	0.1853	0.2522	0.3962	0.3473	**0.2033**
T01	0.0941	0.0692	0.0993	0.1050	0.0749
T02	-0.1119	-0.1316	-0.2466	-0.2237	-0.1087
T180	179.8000	179.7673	179.6452	179.6797	179.8018
T181	-179.7561	-179.7329	-179.6089	-179.6380	-179.7620
T182	179.9918	179.9569	179.9223	179.9379	179.9725
T183	179.4426	179.4135	179.1912	179.2319	179.4542
T184	178.3429	178.3222	177.7812	177.9033	178.4443
T03	15.6606	16.1212	22.3129	21.1998	15.0081
T185	171.2927	171.1579	168.4908	168.9877	171.6515

Table S5: KI

N
C,1,CN
N,2,NC,1,NCN
C,3,C1N,2,C1NC,1,T0,0
C,4,CC,3,CCN,2,T0,0
C,5,C2C,4,C2CC,3,T0,0
O,2,OC,1,OCN,6,T180,0
H,3,H1N,2,H1NC,1,T180,0
H,4,HC,3,HCN,2,T180,0
H,5,H1C,4,H1CC,3,T180,0
N,6,N1C,5,N1CC,4,T180,0
H,11,H2N,6,H2NC,5,T0,0
H,1,H3N,2,H3NC,3,T180,0
Constants:
T0=0.
T180=180.

Param.	rDSD/j3	rDSD	MP2/C3 fc	MP2 C3 ae	rDSD+CV	CC-F12/j3	CC-F12+CV
CN	1.3743	1.3726	1.3728	1.3705	1.3703	1.3731	1.3708
NC	1.3868	1.3863	1.3865	1.3842	1.3840	1.3846	1.3823
NCN	113.6403	113.6479	113.2078	113.2210	113.6611	113.7571	113.7703
C1N	1.3765	1.3747	1.3718	1.3696	1.3725	1.3777	1.3754
C1NC	123.1497	123.1690	123.3387	123.3200	123.1503	123.2012	123.1825
CC	1.3449	1.3440	1.3460	1.3435	1.3415	1.3451	1.3426
CCN	121.7297	121.7321	121.7241	121.7315	121.7573	121.6897	121.6971
C2C	1.4571	1.4559	1.4514	1.4488	1.4533	1.4582	1.4556
C2CC	119.7905	119.7628	119.7818	119.7938	119.7748	119.7052	110.7172
OC	1.2156	1.2136	1.2153	1.2136	1.2119	1.2134	**1.2117**
OCN	123.8348	123.8853	124.1393	124.1329	123.8789	123.7490	123.7426
H1N	1.0060	1.0059	1.0050	1.0043	1.0052	1.0048	1.0041
H1NC	115.3923	115.3323	115.1763	115.1790	115.3350	115.2995	115.3022
HC	1.0817	1.0820	1.0798	1.0786	1.0808	1.0806	1.0794
HCN	115.5736	115.5964	115.7484	115.7655	115.6135	115.6042	115.6213
H1C	1.0795	1.0799	1.0776	1.0764	1.0787	1.0788	1.0776
H1CC	120.8707	120.8782	120.5560	120.5502	120.8724	120.9725	120.9667
N1C	1.2816	1.2800	1.2829	1.2807	1.2778	1.2805	1.2783
N1CC	128.8371	128.8260	129.0964	129.0513	128.7809	128.7378	128.6927
H2N	1.0169	1.0166	1.0161	1.0151	1.0156	1.0159	1.0149
H2NC	110.0014	110.1743	109.0495	109.1747	110.2995	109.8511	109.9763
H3N	1.0103	1.0101	1.0098	1.0089	1.0092	1.0091	1.0082
H3NC	115.6370	115.7437	115.5686	115.5881	115.7632	115.7156	115.7351

Table S6: KIc

N
C,1,CN
N,2,NC,1,NCN
C,3,C1N,2,C1NC,1,T0,0
C,4,CC,3,CCN,2,T0,0
C,5,C2C,4,C2CC,3,T0,0
O,2,OC,1,OCN,6,T180,0
H,3,H1N,2,H1NC,1,T180,0
H,4,HC,3,HCN,2,T180,0
H,5,H1C,4,H1CC,3,T180,0
N,6,N1C,5,N1CC,4,T180,0
H,11,H2N,6,H2NC,5,T180,0
H,1,H3N,2,H3NC,3,T180,0
Constants:
T0=0.
T180=180.

Param.	rDSD/j3	rDSD	MP2/C3 fc	MP2/C3Zae	rDSD+CV	CC-F12/j3	CC-F12+CV
CN	1.3803	1.3795	1.3801	1.3778	1.3772	1.3798	1.3775
NC	1.3809	1.3797	1.3796	1.3773	1.3774	1.3783	1.3760
NCN	113.7320	113.7372	113.2804	113.3073	113.7641	113.8400	113.8669
C1N	1.38081	1.3799	1.3771	1.3748	1.3776	1.3828	1.3805
C1NC	123.1400	123.1418	123.3094	123.2870	123.1194	123.1677	123.1453
CC	1.3432	1.3420	1.3441	1.3415	1.3394	1.3432	1.3406
CCN	121.7413	121.7544	121.7527	121.7552	121.7569	121.6850	121.6875
C2C	1.4545	1.4541	1.4496	1.4470	1.4515	1.4562	1.4536
C2CC	120.0842	120.0642	120.0975	120.1093	120.0760	119.9996	120.0114
OC	1.2162	1.2141	1.2157	1.2140	1.2124	1.2139	1.2122
OCN	123.1987	123.1800	123.4010	123.3844	123.1634	123.0579	123.0413
H1N	1.0060	1.0058	1.0051	1.0042	1.0049	1.0047	1.0038
H1NC	115.4026	115.4070	115.2508	115.2559	115.4121	115.3725	115.3776
HC	1.08129	1.0816	1.0794	1.0782	1.0804	1.0802	1.0790
HCN	115.5019	115.4637	115.6135	115.6319	115.4821	115.4664	115.4848
H1C	1.0783	1.0787	1.0764	1.0751	1.0774	1.0776	1.0763
H1CC	121.7923	121.8357	121.5837	121.5929	121.8449	121.9119	121.9211
N1C	1.2810	1.2797	1.2823	1.2802	1.2776	1.2801	1.2780
N1CC	121.6409	121.6581	121.5488	121.5588	121.6681	121.6078	121.6178
H2N	1.0200	1.0197	1.0195	1.0184	1.0186	1.0191	1.0180
H2NC	111.7923	111.8956	110.8537	110.9918	**112.0337**	111.5824	111.7205
H3N	1.0095	1.0094	1.0091	1.0082	1.0085	1.0084	1.0075
H3NC	113.7330	113.7417	113.4531	113.4655	113.7541	113.7167	113.7291
